# spatialHeatmap: visualizing spatial bulk and single-cell assays in anatomical images

**DOI:** 10.1093/nargab/lqae006

**Published:** 2024-02-02

**Authors:** Jianhai Zhang, Le Zhang, Brendan Gongol, Jordan Hayes, Alexander T Borowsky, Julia Bailey-Serres, Thomas Girke

**Affiliations:** Institute for Integrative Genome Biology, Department of Botany and Plant Sciences, 1207F Genomics Building, University of California, Riverside, CA 92521, USA; Institute for Integrative Genome Biology, Department of Botany and Plant Sciences, 1207F Genomics Building, University of California, Riverside, CA 92521, USA; Institute for Integrative Genome Biology, Department of Botany and Plant Sciences, 1207F Genomics Building, University of California, Riverside, CA 92521, USA; Institute for Integrative Genome Biology, Department of Botany and Plant Sciences, 1207F Genomics Building, University of California, Riverside, CA 92521, USA; Center for Plant Cell Biology, Department of Botany and Plant Sciences, University of California, Riverside, Riverside, CA 92521, USA; Center for Plant Cell Biology, Department of Botany and Plant Sciences, University of California, Riverside, Riverside, CA 92521, USA; Institute for Integrative Genome Biology, Department of Botany and Plant Sciences, 1207F Genomics Building, University of California, Riverside, CA 92521, USA

## Abstract

Visualizing spatial assay data in anatomical images is vital for understanding biological processes in cell, tissue, and organ organizations. Technologies requiring this functionality include traditional one-at-a-time assays, and bulk and single-cell omics experiments, including RNA-seq and proteomics. The *spatialHeatmap* software provides a series of powerful new methods for these needs, and allows users to work with adequately formatted anatomical images from public collections or custom images. It colors the spatial features (e.g. tissues) annotated in the images according to the measured or predicted abundance levels of biomolecules (e.g. mRNAs) using a color key. This core functionality of the package is called a spatial heatmap plot. Single-cell data can be co-visualized in composite plots that combine spatial heatmaps with embedding plots of high-dimensional data. The resulting spatial context information is essential for gaining insights into the tissue-level organization of single-cell data, or vice versa. Additional core functionalities include the automated identification of biomolecules with spatially selective abundance patterns and clusters of biomolecules sharing similar abundance profiles. To appeal to both non-expert and computational users, *spatialHeatmap* provides a graphical and a command-line interface, respectively. It is distributed as a free, open-source Bioconductor package (https://bioconductor.org/packages/spatialHeatmap) that users can install on personal computers, shared servers, or cloud systems.

## Graphical Abstract

**Graphical Abstract:**
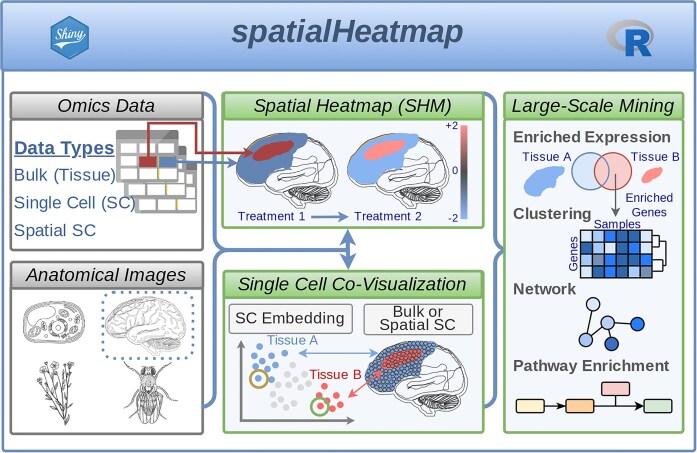

## Introduction

The study of biological systems involves a wide range of spatial measurements for dissecting cell-, tissue- and organ-specific processes (1,2). The corresponding bioassays often cover genetic, molecular, or biochemical profiling methods at low- or high-throughput scales. The latter includes so-called omics technologies widely applied to bulk or single-cell samples collected from specific locations of a tissue or an organism. The technologies used comprise next-generation sequencing or mass spectroscopy for transcript (e.g. RNA-seq) or proteome and metabolome profiling, respectively (3–5). Combined, they enable a new era of spatial biology that provides unprecedented opportunities for studying complex processes in plants, fungi and animals, such as differential responses to the environment, cell differentiation mechanisms, and disease development. Moreover, they offer new directions in medicine and agriculture for precision diagnostics and targeted interventions. Currently, most scientists apply conventional plotting geometries such as scatter, line, or bar plots to encode spatial information in the corresponding legends. However, mapping the information directly onto images containing the anatomical source structures of interest can generate much more realistic representations of spatial information, similar to showing numeric data of regions on geographic maps. While many software tools are available for visualizing quantitative data on geographic maps, spatial plotting resources for anatomical images are limited and need more flexibility. Thus, there is an increasing demand for software allowing the visualization of spatial data in images of the corresponding source structures in an automated, reproducible, extensible, and customizable manner.

Present options for plotting numeric values onto anatomical drawings include electronic *Fluorescent Pictograph Browser* (6,7), *gganatogram* (8), *brainR* (9), *Semantic Body Browser* (10), *TISSUES 2.0* (11) and *Expression Atlas* (12). In addition, single-cell profiling technologies use dimension reduction methods for visualization, such as PCA, UMAP, and tSNE (13–17), and embed the results in scatter plots with cell label information included by coloring the dots accordingly. While helpful in partitioning cells by similarities of assay profiles, embedding plots do not directly reveal spatial or anatomical information. Examples of single-cell analysis software using embedding plots include *Seurat* (18–21), *ASAP* (22), *iSEE* (23), *Single Cell Explorer* (24) and *UCSC Cell Browser* (25).

**Figure 1.**
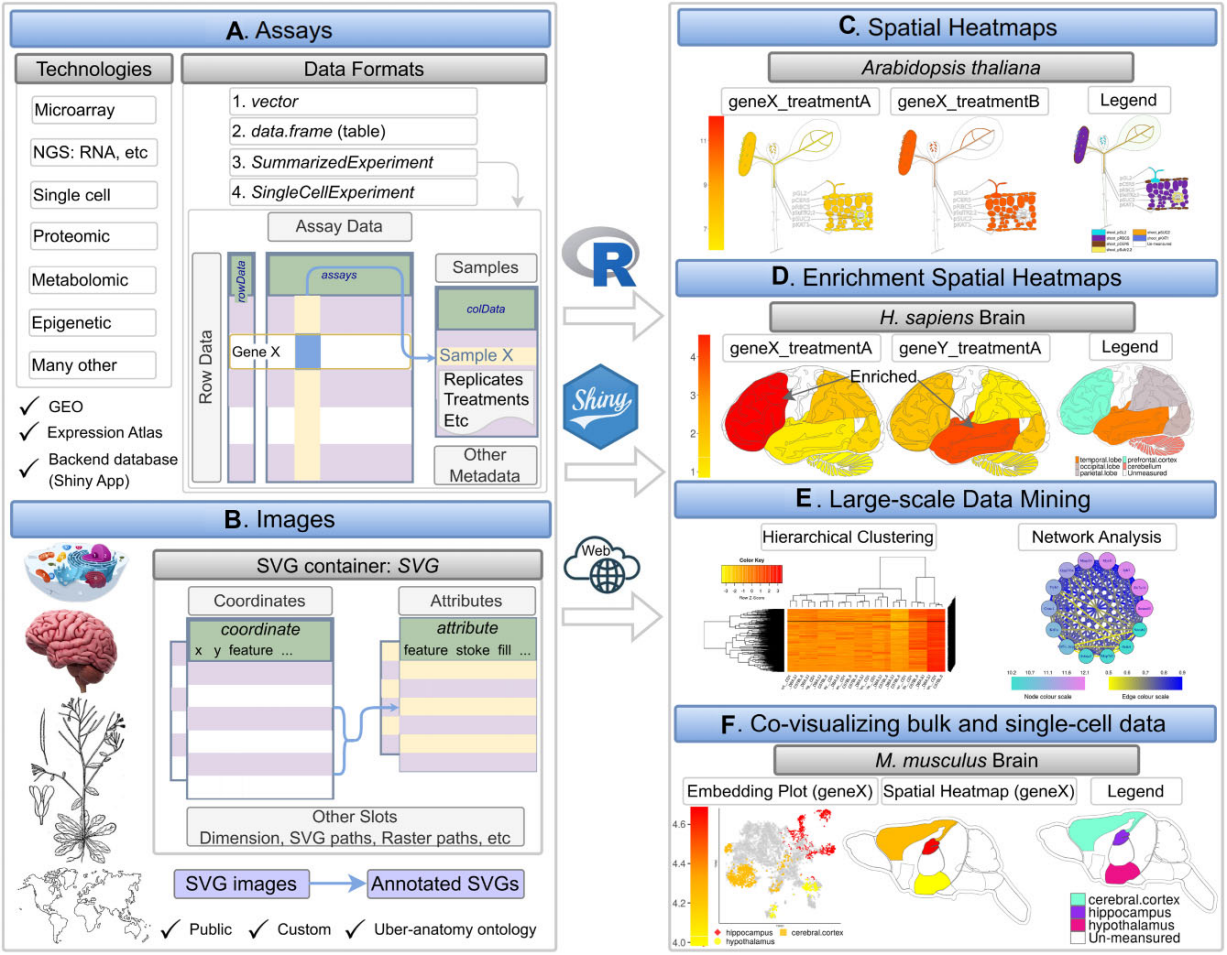
Overview of *spatialHeatmap*. (**A**) The environment stores the numeric assay data in *SummarizedExperiment* or *SingleCellExperiment* objects, and (**B**) imports anatomical images from annotated SVG (aSVG) files into *SVG* objects, an S4 class defined by *spatialHeatmap*. (**C**, **D**) A plotting function generates spatial heatmaps (SHMs) that color the image features by the corresponding assay data. (**E**) Integrating data mining graphics such as matrix heatmaps and network graphs facilitates the identification of molecules with similar abundance profiles. (**F**) Single-cell embedding results can be co-visualized with SHMs.

While existing software applications provide valuable utilities for visualizing spatial data, they lack essential functionalities such as support for (i) using custom anatomical images for generating spatial plots, (ii) co-visualization of anatomical spatial heatmap plots with single-cell embedding plots, (iii) stacking of images with transparency mode to reveal structures at specific layers on demand, (iv) scalability to multiple dimensions such as spatiotemporal data or multiple treatments and (v) graphical and command-line interfaces to support users with different computational skill levels. To address these needs, we have developed the *spatialHeatmap* software (Figure 1) that offers the following core functionalities. First, it visualizes quantitative assay data in images by coloring the corresponding spatial features, such as organelles, cells, tissues, or organs, defined in anatomical images according to a numeric color key. This central feature is called a spatial heatmap (SHM) plot (Figure 1C). The generalized terminology used in this article for referring to image and quantitative assay data is defined in Table 1. The software identifies the spatial features by annotations embedded in the images using the widely adopted Scalable Vector Graphics (SVG) format, referred to as annotated SVGs (aSVGs). Second, when working with large-scale assays, a spatial enrichment function automates the detection of transcripts, proteins, or other biomolecules that are enriched or depleted in certain spatial regions, such as tissue-specific transcripts. Third, cluster and network analysis can identify groups of biomolecules sharing similar assay profiles. Finally, a key feature is integrating tissue and single-cell data by co-visualizing them in composite plots that combine spatial heatmaps with embedding plots of high-dimensional data obtained by various dimension reduction algorithms. Five different methods are provided to generate the tissue-to-cell mappings required for associating SHM with embedding plots: using existing annotations, marker genes, cell clusters, manual assignments, or automated co-clustering of the tissue (bulk) and single-cell data. While related, the latter co-clustering includes several unique steps compared to deconvolution methods for inferring cellular compositions in bulk tissues (26–28). To the best of our knowledge, *spatialHeatmap* is one of the first generic, highly flexible, and object-oriented software tools for visualizing spatial assay data in anatomical images that can be integrated with single-cell embedding plots. Moreover, *spatialHeatmap* can be used either in a command-driven mode from within R or a graphical user interface (GUI) provided by a Shiny application.

**Table 1.** Terminology for spatial data

Term	Description
Spatial features	Spatial elements in SVG images including cells, tissues and organs
Spatial assays	Low- or high-throughput spatial profiling, technologies, including most omics technologies
Biomolecules	Biological molecules quantified by spatial assays, such as mRNAs, proteins and metabolites
Abundance values	Quantity of biomolecules measured by assays, such as gene expression levels

## Materials and methods

### Implementation overview

We implemented *spatialHeatmap* as an R package freely available from Bioconductor. The software uses generic S4 classes to manage image and bioassay data. It takes advantage of existing classes within the Bioconductor software ecosystem and defines new S4 classes where needed. This open design makes the *spatialHeatmap* reusable, extensible and maintainable.

### Data structures

**Figure 2.**
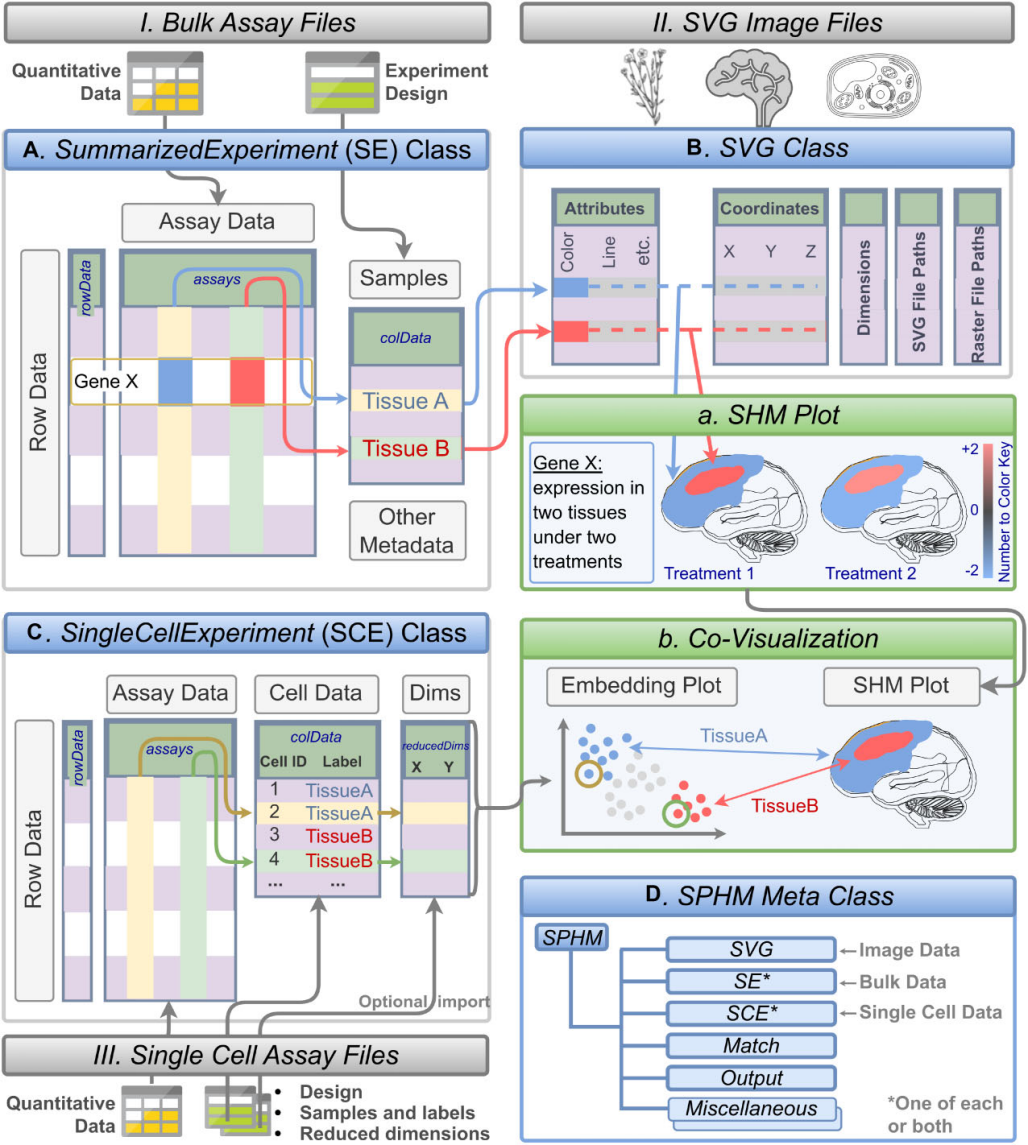
Schematic view of data structures, and creation of SHM and co-visualization plots. File imports, classes, and plotting functionalities are illustrated in boxes with color-coded title bars in grey, blue and green, respectively. Quantitative and experimental design data (I) are imported into matching slots of an *SE* container (**A**). aSVG image files are stored in *SVG* containers, an S4 class developed by this project (**B**). Expression profiles of a chosen gene (GeneX) in (A) are mapped to the corresponding spatial features in (B) via common identifiers (here TissueA and TissueB). The assembled information is used to plot an SHM (A) where the quantitative data is represented in the matching features by colors according to a number to color key. For co-visualization plots, single-cell data can be imported as well, which is stored in the *SCE* object class (**C**). Reduced dimension data for embedding plots can be generated on the fly or imported from files. The single-cell embedding results are co-visualized with SHMs where the cell-to-tissue mappings are indicated by common colors in the co-visualization plot (B). The *SPHM* meta class (**D**) organizes the individual objects (A–C) along with internally generated data.

The package organizes the quantitative and experimental metadata of bulk and single-cell assays in *SummarizedExperiment* (*SE*) and *SingleCellExperiment* (*SCE*) containers (29,30), respectively (Figure 1A and Figure 2A–C). They are two of the core S4 classes within the Bioconductor ecosystem widely used by many other software packages operating on various omics data. In the case of gene expression data, the *assay* slot of the two containers stores them in a matrix-like structure, where the rows and columns represent the genes and samples, respectively. In contrast, the *colData* slot (Figure 2A) contains sample metadata such as replicate information. The tissue or cell type information in these objects maps via *colData* to the corresponding spatial features in the aSVG images using unique identifiers. The mapping allows coloring the elements of interest in an aSVG image according to the numeric assay data. For simplicity, users can also provide the numeric data in a numeric *vector* or *data.frame*. Supporting these simple data structures is helpful for low-throughput assays.

SVG was selected as the main image format in *spatialHeatmap* for several reasons. First, it is a widely adopted open community standard developed by the World Wide Web Consortium. Second, its XML-based text format makes it computationally easy to modify image elements. Third, it supports layering of vector and photographic images with transparency support. Fourth, it supports interactive features and animations. Fifth, various vector graphics editing software packages are available for generating and editing SVG images (e.g. Inkscape or Adobe Illustrator). For plotting SHMs, the spatial features of interest need to be annotated in the SVG files. The resulting annotated SVGs (aSVGs) are imported into R where they are stored in an *SVG* container, an S4 class defined by *spatialHeatmap* (Figures 1B and 2B). Its *coordinate* slot stores the coordinates of spatial features, while the *attribute* slot defines the styling of these features, such as color and line width. Other slots include *dimension*, *svg* and *raster*, corresponding to width and height, aSVG file paths, and raster image path, respectively. Raster images are required only when including photographic image components in SHMs, which is optional. Various methods were developed to access, edit, and create *SVG* object instances, such as subsetting spatial features, changing style attributes, and combining *SVG* instances. In addition, a *SPHM* meta container was introduced to organize individual data and image objects needed for plotting *SHMs*, and optionally integrate them with single-cell data (Figure 2D). This list-like S4 class stores the data and image objects in dedicated *bulk*, *cell*, and *svg* slots. Other components of this class link spatial features with the corresponding cell types, and store the graphics instructions for SHMs and co-visualization plots.

### Custom data and public repositories

*spatialHeatmap* supports the usage of custom assay and image data. Alternatively, users can work with data downloaded from public repositories. Spatial assay data are available in a wide range of repositories, such as GEO (31) and Expression Atlas (12). Compatible SVG and aSVG image data are available in the GitHub repositories *EBI anatomogram* and *SHM aSVG* that are maintained by the EBI Gene Expression Group and this project, respectively. The latter allows users to contribute custom aSVG files, as described in the accompanying *spatialHeatmap* tutorial for creating aSVG files (linked from the vignette on Bioconductor).

### Spatial heatmaps

The data structures and processing steps for generating *SHMs* are illustrated in Figure 2A, B. First, the required quantitative assay, experimental design, and image data (Figure 2I–III) are imported from files and stored in the corresponding *SE* (Figure 2A), *SVG* (Figure 2B), and *SCE* (Figure 2C) objects, respectively. After importing the image and assay data, users can plot SHMs (Figure 2A). The *SVG* and *SE* (or *SCE*) objects contain the anatomical structures and assay information to generate plots, respectively. The coloring of the spatial features (e.g. tissues) is proportional to the numeric assay values. Users can look up the meaning of the colors in a numeric color key included in the legend of the SHM plots. Further, the environment establishes proper feature-to-sample associations via common identifiers or a mapping table. The usage of well-established layer-based graphics environments, such as *ggplot2* and *gridExtra* (32), enables the integration of multiple plot features, while simplifying future extensibility. The following provides examples of important features included in the current release version of the software. First, a flexible layout option allows the arrangement of several SHMs next to each other. This feature helps compare the abundance profiles across biomolecules (e.g. mRNAs or samples (e.g. treatments). Second, in the case of multi-layer images, the software supports transparency to visualize features of several layers simultaneously or hide elements in top layers to expose those in lower layers. Third, the SHM layout scales to complex experimental designs with many dimensions, such as spatiotemporal designs with several treatment variables. Fourth, users can generate photographic SHMs by superimposing line drawings on raster graphics. Fifth, interactive SHMs can be generated as well as videos (33). Sixth, SHMs can be plotted using either absolute or relative abundance values. The latter can be *log*_2_ fold changes calculated for a treatment relative to a control sample.

### Large-scale data mining

SHMs are a powerful visualization tool for focused analyses routines comparing assay profiles among smaller numbers of biomolecules across cell types and conditions. This is the case because each biomolecule requires its own SHM plot. To support analysis routines involving large numbers of different biomolecules, we have integrated functionalities for large-scale data analysis. These functionalities include *Spatial Enrichment* for identifying groups of biomolecules with similar or opposite assay profiles. Data mining methods, such as clustering and network analysis, are also included that scale to much larger numbers of biomolecules than SHMs.

#### Spatial enrichment

The primary utility of *Spatial Enrichment* is the automated detection of groups of biomolecules with enriched or depleted abundance in the profiled spatial features, and subsequently visualize these patterns in SHMs. Common use cases include the identification of differentially expressed genes (DEGs) with tissue-specific expression patterns or those with uniform expression across tissues. For detecting them, the software supports systematically identifying spatially enriched or depleted biomolecules that are significantly up or down regulated in one feature relative to the other features of interest. The stringency of this approach can be relaxed by allowing a user-definable number of outlier features. The statistical methods for detecting spatial DEGs include *edgeR* (34), *limma* (35) and *DESeq2* (36). The enrichment results among spatial features can be compared in overlap tables, Venn diagrams and *UpSet* plots (37). In addition, the identified spatial DEGs can be visualized in enrichment SHMs where features not involved in the comparisons can be set transparent (Figure 1D). *Spatial Enrichment* can also be used for detecting treatment-specific DEGs by similar all-against-all comparisons.

#### Matrix heatmap and network graphs

*spatialHeatmap* uses clustering and network analysis to identify groups of biomolecules sharing similar abundance profiles and visualize the obtained clusters in matrix heatmaps or network graphs. The assay matrix component in *SE* provides the data for the other biomolecules participating in these large-scale analyses. Integrating these methods into *spatialHeatmap* helps identify for a biomolecule plotted in an SHM all other biomolecules with similar abundance patterns. The package supports several similarity metrics to identify biomolecules with similar expression patterns, such as correlation coefficients and distance methods. To rank or filter the results, users can specify fixed numbers of genes, percentage values, or similarity threshold values. After identifying a group of biomolecules with similar abundance patterns, the environment applies clustering (e.g. hierarchical or K-means clustering) to the corresponding rows in the matrix (32,33). The result is displayed as a matrix heatmap (Figure 1E, Figure 4) where the rows and columns are sorted according to the corresponding hierarchical clustering dendrograms. Moreover, static and interactive viewing options are provided, including zooming functionalities. Network analysis can be performed using *WGCNA* (38), *flashClust* (39) and *dynamicTreeCut* (40). The objective is to identify network modules that can be visualized in the form of network graphs. The network modules represent groups of genes sharing highly similar expression profiles. The network graphs provide the same static and interactive viewing options as the matrix heatmaps above. The nodes and edges in the network graphs represent biomolecules and adjacencies between nodes, respectively (Figure 4C). The thickness of the edges denotes the adjacency levels, while the size of the nodes indicates the degree of connectivity among the biomolecules. Supplementary Section S1 provides additional details about network analysis in *spatialHeatmap*.

**Figure 3.**
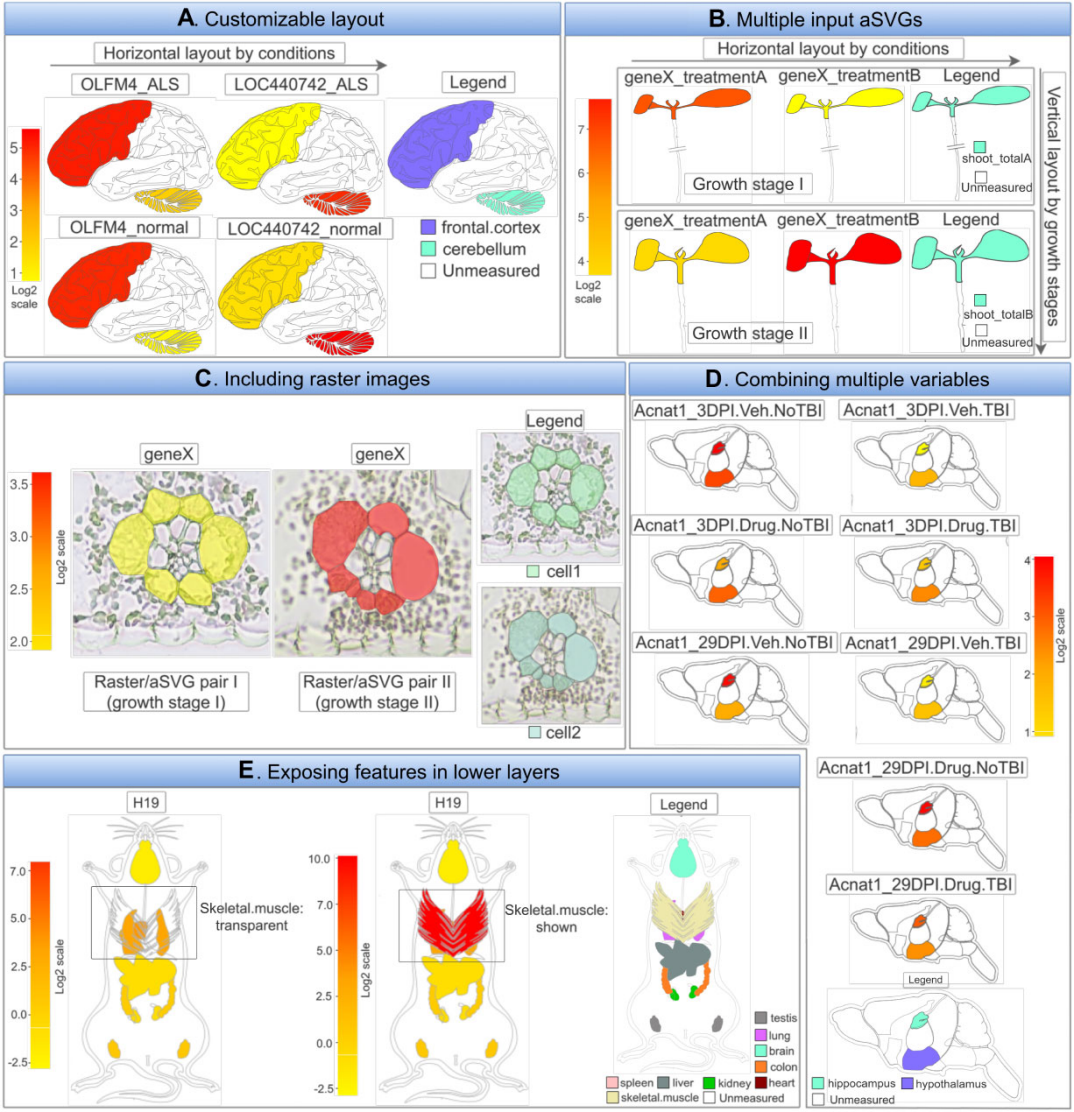
Selected examples showcasing the SHM plotting functionalities of *spatialHeatmap*. (**A**) *Layout example for multiple biomolecules and conditions*. Four SHMs are plotted for human brain, each containing one cross-section. The experimental variables, genes (*OLFM4* and *LOC440742*) with and without disease (ALS) are arranged row- and column-wise, respectively. The number-to-color key (left) and the legend plot (right) indicate the gene expression levels in the corresponding tissues. (**B**) *Assembling SHMs from separate aSVG input files*. This option provides flexibility for joining SVG images on the fly without changing the source image files. (**C**) *Photo-realistic effect by combining SVG with raster images*. An example is shown where the SVG line graphics has been superimposed onto matching raster images to show additional structural details. The visibility of the spatial features in the line graphics is maintained by increasing the transparency level of the raster image. (**D**) *Many experimental variables*. Example demonstrating the scalability of the SHM layout functionalities using many experimental variables. (**E**) *Transparency reveals features in lower layers*. In this example skeletal muscle tissue obstructs the view onto organs in a lower layer. Increasing the transparency in the upper layer reveals other organs, here heart and lung.

**Figure 4.**
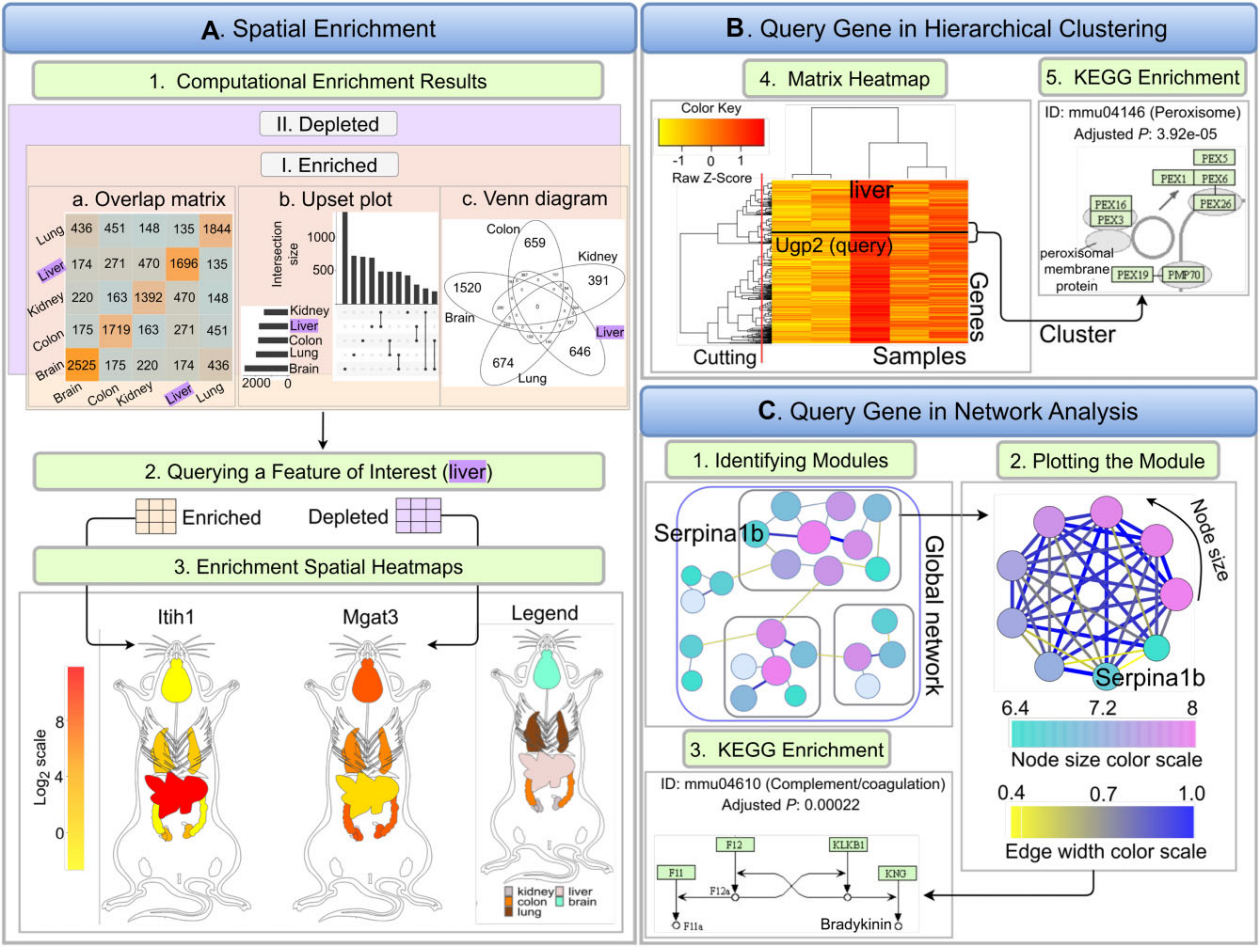
Extensions for large-scale data. (**A**) Five spatial features are chosen as examples (brain, lung, liver, colon, kidney) for spatial enrichment analysis. Spatially enriched or depleted genes in each spatial feature relative to three other features are detected, while allowing one outlier. Intersects among biomolecule sets can be plotted as intersect heatmaps, UpSet plots or Venn diagrams (A1). Liver is used as query feature, and the corresponding enriched and depleted genes are returned in separate tables (A2). Next, one enriched (*Itih1*) and one depleted gene (*Mgat3*) are chosen for plotting enrichment SHMs (A3). (**B**) Hierarchical clustering is performed to detect genes with similar expression profiles and the results are presented in a matrix heatmap where *Ugp2*, another liver-specific gene, is highlighted by a horizontal line (B1). KEGG enrichment reveals that this cluster is enriched in genes involved in the Peroxisome pathway (B2). (**C**) A global network is constructed and partitioned to generate separate gene modules (C1–C2). The module containing the query gene *Serpina1b* is retrieved and plotted. Nodes and edges in the graph represent genes and adjacencies, respectively.

To functionally interpret gene clusters, enrichment analysis of functional terms can be performed (41). Several functional annotation systems are supported, such as Gene Ontology (GO) and KEGG pathways (Kyoto Encyclopedia of Genes and Genomes). These annotations are based on Bioconductor annotation packages that are regularly updated and tested by Bioconductor’s biannual release cycle. This ensures that the underlying annotations continue to be up-to-date.

### Co-visualization

The *spatialHeatmap* package provides novel functionalities to integrate tissue and single-cell data by visualizing them in composite plots that combine SHMs with embedding plots of high-dimensional data (Figure 2B). Identical colors indicate matching components between SHM and embedding plots. The resulting spatial context information provides insights into the tissue-level organization of single-cell data, or vice versa. For co-visualizing single-cell data with tissue features, the individual cells of the single-cell data map via their group labels to the corresponding tissue features in an *SVG* instance initialized from an aSVG file (TissueA and TissueB in Figure 2B and C). A translation map can be used to avoid manual relabelling if the feature labels in an aSVG are different than the corresponding cell group labels. The assay data and cell group labels are stored in the corresponding *SE* (Figure 2A) and *SCE* (Figure 2C) object slots, respectively (30). Finally, *spatialHeatmap* supports several dimensionality reduction algorithms including PCA (13,14), UMAP (15,17) and tSNE (16,17) to generate single-cell data embedding plots.

The co-visualization module provides five distinct methods for generating the tissue-to-cell mappings required to associate SHMs with embedding plots. These methods include the use of existing annotations, marker genes, manually assigning labels, or by co-clustering of the bulk tissue and single-cell RNA-seq data. Annotation and manual assignment methods obtain the cell labels in different ways. While the annotation-based method uses existing group labels stored in the metadata of single-cell data (*e.g*. single-cell RNAseq), the manual method allows users to create custom cell-to-tissue associations one-by-one or import them from tabular files. The manual method offers the highest level of flexibility and includes the generation of group labels with clustering algorithms for single-cell data (14,17,30,42). In contrast, the automated method uses a co-clustering algorithm to assign source tissues to the corresponding single cells computationally (13,15,16,42). This co-clustering is experimental and requires bulk expression data from the tissues represented in the single-cell data. Supplementary Section S2 provides additional details about this method.

The co-visualization plots (Figure 2.2) indicate matching cells and tissues by using identical colors in the embedding plots and SHMs, respectively. The colors can represent any type of custom or numeric information. In a typical use case, either fixed tissue-specific colors or a heat color gradient is used that is proportional to the numeric expression information obtained from the single-cell or bulk expression data of a chosen biomolecule. When the expression values among groups are very similar, toggling between the two coloring options is important to track the tissue origin in the single-cell data. To color by single-cell data, one often wants to first summarize the expression of a given gene across the cells within each group via meaningful summary statistics, such as mean or median. Cells and tissues with the same group label will be colored the same (here by abundance value). To color by tissues, the color used for each tissue feature will be applied to the corresponding cell groups represented in the embedding plot. Finally, cell-by-value coloring can be applied to view the abundance levels of biomolecules within each cell and tissue.

### Graphical user interface using Shiny

The functionalities of *spatialHeatmap’s* command-driven R version can be accessed from the graphical user interface (GUI) of *spatialHeatmap Shiny*. Its intuitive-to-use interface targets non-expert users, including experimentalists who are not familiar with R. *spatialHeatmap Shiny* can run from desktop computers or centralized web or cloud services. The latter option includes shinyapps.io, Amazon Web Services, Google Cloud Platform and Azure. A test instance of *spatialHeatmap Shiny* is deployed on shinyapps.io (see URL in Availability Section). The Shiny App allows users to create customized instances with options to include custom sample data (numeric assay data and aSVG images) and to set styling defaults. For details, users want to consult the Shiny section in the vignette of the *spatialHeatmap* package.

### Backend database

For optimal scalability and performance, *spatialHeatmap*’s R and Shiny versions can load the assay data from an HDF5 backend database that stores the relevant assay data and aSVG files. This is useful when working with large and complex assay data. For populating the database, accepted data formats include *data.frame*, *SE* and *SingleCellExperiment*.

## Results

The following illustrates the most important functionalities of *spatialHeatmap* covering: (i) SHM plots with different configurations and multi-layer viewing options; (ii) functionalities for creating matrix heatmaps and network graphs; (iii) co-visualizing single-cell data together with SHMs and (iv) a use case example demonstrating the usefulness of *spatialHeatmap* for gaining novel insights into cellular mechanisms.

### SHM plots

Users can generate SHM plots with a single generic plotting function (*shm*) or using the graphical interface of the associated Shiny application (see below). The flexible design of this function supports many default layouts and customization needs for plotting SHMs. Several common plotting scenarios are introduced below with illustrations given in Figure 3.

#### (A) Customizable layout

When plotting SHMs, a common requirement is to compare the abundance profiles of multiple biomolecules (e.g. mRNAs) across treatments and tissues. The plotting utilities in *spatialHeatmap* can generate the SHM layouts required for these comparisons automatically or based on custom layout parameters provided by the user. To demonstrate this functionality, Figure 3A gives an example of SHMs for two genes and two treatments, here laid out in horizontal and vertical directions, respectively. Each SHM includes one cross-section obtained from human brain. A legend plot on the side defines the features shown in a SHM. The orientation of the layout of multiple SHMs can be changed as needed. Importantly, the layout utility extends to large numbers of experimental variables in both dimensions. This flexibility and scalability in organizing multiple SHM plots is particularly useful when working with larger numbers of genes or conditions, such as members of a complex gene family and multiple diseases at different time points. The specific numeric expression data chosen for this example are the mRNA abundance levels of the *OLFM4* and *LOC440742* genes from an RNA-seq experiment of human cerebellum and frontal cortex tissues with or without amyotrophic lateral sclerosis (ALS) (43). The assay data and aSVG image were downloaded from the Expression Atlas (12) and EBI’s anatomogram repository, respectively.

Apart from flexible layout and legend parameters, the environment allows users to customize many additional options, such as the overall color scheme, font styles, and the appearance of the legend plot and the number-to-color key.

#### (B) Multiple input aSVGs files

In Figure 3A, SHMs are created from a single input aSVG image file. Other use cases require support for generating SHM plots from multiple aSVG files. This functionality is also supported by *spatialHeatmap*. Organizing the images of different development stages of a spatiotemporal assay in separate aSVG files can be more flexible by enabling the rearrangement of images on the fly as part of the SHM layout process rather than using static multi-component images where rearrangments require time consuming manual changes to the source image. Figure 3B gives an example of this functionality where an SHM view is assembled from two aSVGs files, here representing different developmental stages and treatments of *Arabidopsis thaliana* seedlings (44). Another application based on multiple aSVG files is the visualization of assay profiles from orthologous genes across spatial images from different species (not included in Figure 3), where the individual species are plotted next to each other and the corresponding source images are stored in separate aSVG files.

#### (C) Photo-realistic views with raster images

SVG images most suitable for generating SHMs are easy to interpret line drawings clearly defining the features that will be colored by the corresponding quantitative assay data. Including at the same time photo-realistic details reduces clarity in SHM plots. However, in certain use cases it is important to add photo-realistic details of complex structures. To address this need, *spatialHeatmap* supports superimposing SVG line drawings onto raster images without the need to change the orginal aSVG images. The visibility of the spatial features in the line drawings can be largely maintained by adjusting the transparency of the raster images at user-definable levels. An additional option is to incrementally reduce color to black and white. In Figure 3C, matching vector and raster images have been superimposed representing a cross-section of maize leaves (45), where the expression level of a chosen gene is illustrated by the coloring in the corresponding spatial features accordingly.

#### (D) Many experimental variables

As mentioned under subsection A, the SHMs plots of the *spatialHeatmap* software scale to large numbers of experimental variables. A more complex example of this functionality is showcased in Figure 3D. The data used for this example are the expression levels of the *Acnat1* gene from an RNA-seq experiment of mouse brain after traumatic brain injury (TBI) (46). Four experimental variables are considered in this example: two spatial features (hippocampus and hypothalamus), two time points (3 or 29 days post injury), TBI or sham injury, and drug *versus* control treatments. Eight SHMs with different combinations of experimental variables are compared in Figure 3D.

#### (E) Exposing features from lower layers

Complex anatomical SVG images with multiple layers often contain spatial features that overlap with each other from specific viewpoints. To reveal hidden features in lower layers, one can apply adjustable transparency levels to the upper layer(s). This functionality is illustrated in Figure 3E, where the skeletal muscle tissue obstructs the view onto the organs in the lower layer (panel E middle). After making the upper layer transparent, lung and heart tissues become visible in the lower layer (panel E left).

### Integrated large-scale analysis

Since SHMs are particularly useful for analyzing limited numbers of biomolecules (e.g. transcripts) in parallel, we have included large-scale analysis methods in *spatialHeatmap*. With these extensions users can integrate their targeted spatial analysis results with the other biomolecules included in their assay tables which are often large-scale genome-wide data sets (Figure 2A). By combining these data users can systematically identify clusters of biomolecules sharing positively or negatively correlated abundance profiles with the biomolecules used in their SHM analysis as well as the corresponding network modules. Another application is the systematic identification of biomolecules that are enriched or depleted in certain spatial features.

#### Spatial enrichment

Given a group of spatial features, a spatial enrichment analysis identifies abundance differences of biomolecules among spatial features. Biomolecules up- or down-regulated in one spatial feature relative to other features are denoted spatially enriched or depleted, respectively (Figure 4A.1). Subsequently, the spatially enriched biomolecules can be visualized in the form of enrichment SHMs (Figure 4A.2) for exploring their spatially selective abundance patterns (Figure 4A.3).

The enrichment functionality is demonstrated (Figure 4A) with mouse RNA-seq data from a study comparing organ-level gene expression patterns across mammalian species (12,47). The corresponding aSVG file is from EBI’s anatomogram repository. Five spatial features are considered, including brain, liver, lung, kidney, and colon. For demonstration purposes, outliers are allowed to assure some degree of overlap of genes in the enrichment results of the chosen organs. Overlaps among sets of biomolecules can be represented with three different types of plots: overlap heatmap (matrix), UpSet plot and Venn diagram (Figure 4A.1). Next, liver is chosen as query example for identifying biomolecules with liver-specific abundance patterns (Figure 4A.2). Among them are the mRNAs of the *Itih1* and *Mgat3* genes that are enriched and depleted in liver, respectively. The corresponding abundance values are used to create enrichment SHMs (Figure 4A.3) illustrating that, relative to the other organs, *Itih1* and *Mgat3* show the highest and lowest expression in liver, respectively. Interestingly, both genes show similar abundance in brain and colon, two very distinct organs.

#### Hierarchical clustering

The hierarchical clustering module is designed to integrate SHM analysis with large-scale data mining. A frequent requirement is to identify for a biomolecule represented in an SHM (here query) nearest neighbors that share similar abundance patterns with the query. To address this need, users can use the abundance values of the query in a correlation-based search and subset the assay matrix by similar abundance patterns accordingly (Figure 2A). Next, hierarchical clustering is performed on the subsetted matrix with the query biomolecule included, and the results are presented in the form of a matrix heatmap (Figure 4B.1) where the rows and columns are sorted by the corresponding dendrograms for biomolecules and conditions, respectively. Figure 4B.1 illustrates this functionality using the same RNA-seq data (12,47) as the spatial enrichment step (Figure 4A). The query gene used in this example is *Ugp2* from mouse that is spatially enriched in liver. In the obtained hierarchical clustering heatmap the query gene is indicated by a black line. To obtain a group of genes (cluster) the query is part of, the row dendrogram can be partitioned with a tree-cutting function (48). Alternatively, clusters of interest can be visually inspected via a zooming feature. To functionally interpret the genes in a cluster, enrichment analysis of functional terms, such as KEGG pathways, can be performed using functionalities of the *clusterProfiler* package (41). In the enrichment result of the chosen example (Figure 4B.2), the Peroxisome pathway (mmu04146) from KEGG is the most highly enriched category (Figure 4B.2, adjusted *P-value* =3.92*e* − 05). Peroxisomes are involved in many important metabolic processes including lipid metabolism in hepatocytes (49). This is in alignment with the fact that the query gene *Ugp2* is spatially enriched in liver. Thus, it is reasonable to hypothesize that the less characterized genes of the same cluster (Supplementary Table S1) might also be involved in liver-specific processes.

#### Network analysis

The network analysis module (Figure 4C) implemented in *spatialHeatmap* can be used in combination with the above hierarchical clustering step or as an independent large-scale mining method. It is particularly effective in identifying network modules of biomolecules that are tightly connected by sharing similar abundance profiles among each other including an optional query biomolecule (38–40). To run this analysis, a global network is constructed for the biomolecules stored in the assay matrix that is subsequently partitioned into network modules (Supplementary S1). If needed the assay matrix can be subsetted prior to the network analysis using various methods including the similarity-based approach introduced under the hierarchical clustering step. In the module identification process, biomolecules with highly similar abundance patterns are assigned to the same module. Finally, the genes included in the network modules can be analyzed by functional enrichment analysis of pathway or gene ontology categories. The network result shown in Figure 4C is based on the same input assay matrix used in the hierarchical clustering example (Figure 4B) where *Serpina1b* was chosen as a query. The network module containing the query is plotted as a network graph (Figure 4C.2). Similar to the matrix heatmap, the network graph viewer can be used in a static or interactive mode. Nodes and edges in the graph represent genes and adjacency values between genes, respectively. The node size is proportional to the connectivity which is the sum of a gene’s adjacency values with all other genes in the same module. Typically, the node with the largest size and the thickest edge play the most important roles in the module. Since *Serpina1b* is spatially enriched in liver (not shown), its gene module (Figure 4C.2, Supplementary Table S2) is most likely involved in liver-specific processes. The functional enrichment analysis results for this example confirm this assumption, since liver is known to express the majority of complement proteins (50) and the Complement and Coagulation Cascades pathway (mmu04610) is most significantly enriched in this module (Figure 4C.3; adjusted *P*-value = 0.00022).

### Co-visualization

**Figure 5.**
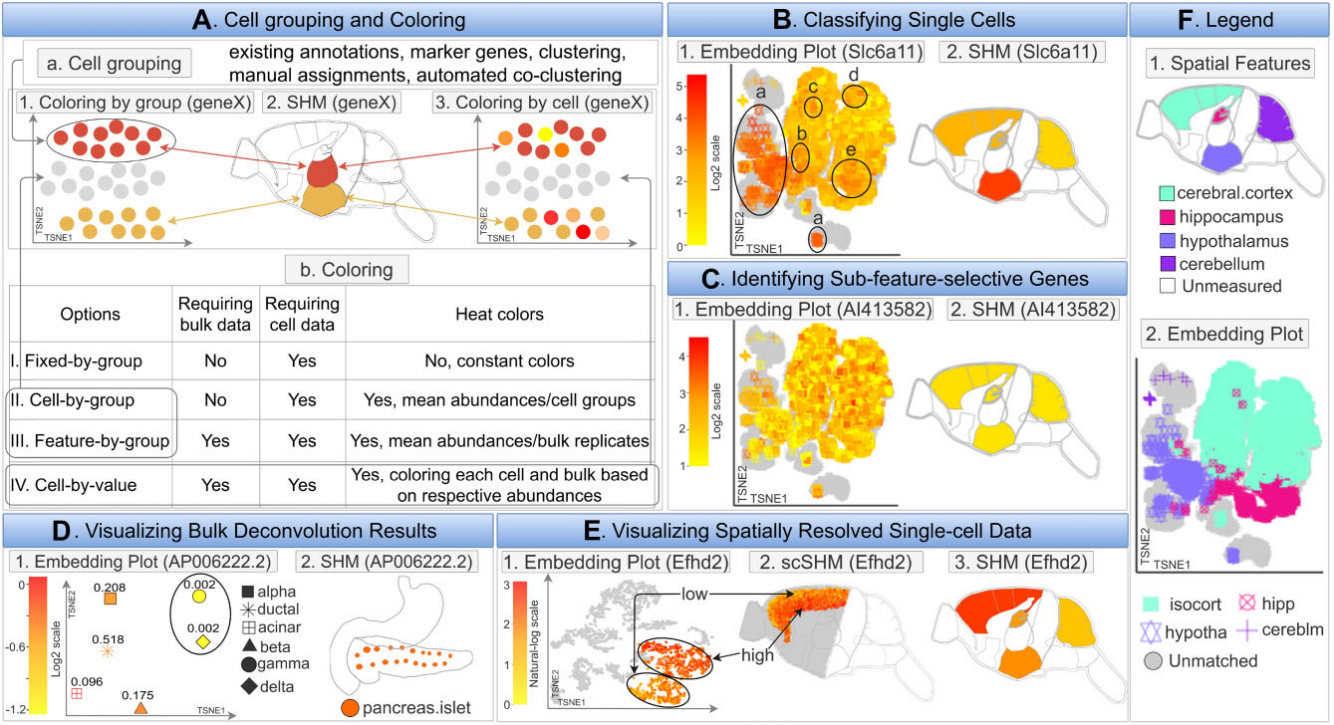
Co-visualizing SHMs with single-cell embedding plots. (**A**) Single-cell data require group labels to visually associate them with the corresponding features in SHMs by colors, see panel Aa. Four coloring schemes are summarized under Ab. The cell groupings and coloring schemes are illustrated under A1-3. (**B**) An example is given for associating cells with source features. The *Slca11* gene is spatially enriched in hypothalamus tissue of mouse brain. The *cell-by-value* heat coloring scheme is used to visualize the expression of *Slca11* in cells and features under B1 and B2. F provides the key for matching cells and features. Cells labeled with Ba-e are associated with hypothalamus tissue. (**C**) An example is given for identifying cells from sub-features. Gene *AI413582* is not detected as spatially enriched or depleted by the *Spatial Enrichment* analysis (C2). However, the co-visualization plot using *cell-by-value* coloring indicates that this gene may be selectively expressed in potential sub-features of the cerebral cortex tissue as indicated by a sub-population of cells with expression patterns that are distinct from the other cells of the same tissue (C1). (**D**) The co-visualization module can also be used for visualizing bulk deconvolution results. In the given example, RNA-seq data of human pancreas islets is deconvolved using single-cell data as references. Bulk tissues (D2) and estimated cell types with proportions (D1) are co-visualized using the expression of *AP006222.2* for heat coloring. The pancreas islets consist of two rare (circled) and four major cell types (D1). (**E**) The co-visualization feature of *spatialHeatmap* can also plot spatially resolved single cell data. In the given example, spatially resolved single-cell RNA-seq data of mouse sagittal brain are co-visualized with four tissues (F.1). The expression values of *Efhd2* are used for coloring. This gene is spatially enriched in the cerebral cortex (E3). The cerebral cortex cells are positioned according to their spatial coordinates in the corresponding scSHM plot (E2). The plot shows that *Efhd2* is selectively expressed in sub-features of the cerebral cortex, which is consistent with the embedding plot (E1). (**F**) Legend plot defining the identity and matching of cells and spatial features.

*spatialHeatmap’s* co-visualization module provides several novel plotting functionalities designed to gain insights into tissue-level organizations of single-cell data, or *vice versa* cellular compositions of tissues (Figure 5B–E). It combines SHM and embedding plots where matching tissues and cells can be associated by identical point colors or shapes. The coloring of the single-cells (dots) and tissue features can be based on quantitative values (heat coloring) or fixed group-based colors (Figure 5A.b). The cell-to-feature matching is established by shared group labels that can be stored in the metadata slots of the corresponding data objects (Figure 2C). The group labels of cells can be imported or generated by the software (Figure 5A.a). This includes support for existing cell annotations, marker gene-based methods, clustering, manual assignments, and co-clustering of bulk and single-cell data (Supplementary S2). The following showcases several important functionalities of the co-visualization tool. The data used in these examples (Figure 5B, C) includes single-cell (51) and bulk (52) RNA-seq data from multiple tissues of mouse brain. The tissues represented in both bulk and single-cell data are cerebral cortex (isocortex), hippocampus, hypothalamus, and cerebellum (Figure 5B, C).

#### Coloring options

Examples of the most important coloring options are given in Figure 5A.b. First, *fixed-by-group* coloring assigns to each tissue a specific color in the SHM that will also be used for coloring the corresponding cells in the embedding plot. Colors can be automatically assigned to features or selected by the user. Second, *cell-by-group* colors cells and features by a user definable summary statistics. In Figure 5A.1-2, the single-cell abundance values of a gene were averaged within groups of cell types, and then used for coloring the corresponding cells and features in the embedding and SHM plots, respectively. Orphan cells lacking feature assignments are presented in grey. This coloring mode is useful for assessing whether the tissue-level summarized single-cell data agree overall with the expression trends of the bulk data from the same tissues. Alternatively, it can be used as quality control for approximating potential bias in the cell populations collected from the individual tissues. *Feature-by-group* coloring is similar, but assigns colors based on bulk abundance data. Third, *cell-by-value* coloring generates the most detailed heat coloring by assigning a specific abundance value to each cell, e.g. obtained from a chosen biomolecule (Figure 5A.3, Table 1). Similarly, the features in the SHM plot are colored by the abundance of the same biomolecules. If additional coloring methods are needed, users can customize the coloring scheme.

#### Single-cell classification via marker biomolecules

The co-visualization functionality can be used to visually associate single cells with their source features via marker biomolecules (Figure 5B). For this, one can generate a co-visualization plot where the abundance of a known feature-specific biomolecule is used for *cell-by-value* coloring. Cells and features can be assigned to each other if their abundance patterns of a chosen marker molecule are similar. Figure 5B.2 gives an example for mouse brain. In this case the *Spatial Enrichment* module has identified *Slc6a11* as spatially enriched in hypothalamus tissue. Cells with similar expression of *Slc6a11* are visualized in the embedding plot of Figure 5B.1 with the *cell-by-value* coloring scheme. The matching between tissues and single cells is given in Figure 5F. In this example, cells labelled a–e can be associated with hypothalamus based on the expression similarity of *Slc6a11* in the SHM (Figure 5B.2). Additionally, cells from different tissues (known from available cell annotations) with similar expression of the chosen marker gene may represent the same or similar cell types present in different source tissues (e.g. blood vessels).

#### Identification of sub-feature-selective genes

The spatial enrichment analysis of bulk data might fail to detect biomolecules that are only differentially expressed in sub-features of a chosen tissue, e.g. due to sensitivity limitations of an assay technology. To overcome this obstacle, the co-visualization functionality can be used. Figure 5C shows a co-visualization for gene *AI413582* that is not detected as spatially enriched in any of the assayed tissues (Figure 5C.2). The matching between tissues and single cells is given in Figure 5F. The embedding plot with *cell-by-value* coloring (Figure 5C.1) reveals that this gene is only selectively expressed in a sub-population of cerebral cortex (isocortex) cells.

#### Visualization of bulk deconvolution results

Many deconvolution algorithms have been developed to infer cellular compositions in bulk data (27,53–56) that can be visualized using the co-visualization module. Specifically, the cell type proportions estimated by the deconvolution methods will be used to calculate an expression matrix from the bulk data where rows are genes and columns are inferred cell types. Next, the cell type expression matrix and the bulk assay data will be used for co-visualization (Figure 5D). This utility is showcased using *MuSiC* (56) for deconvoluting bulk RNA-seq data from human pancreatic islets (57) with matching single-cell data (58). Figure 5D.2 shows the SHM of pancreatic islets for gene *AP006222.2* and Figure 5D.1 gives the corresponding embedding plot of the inferred cell types, where *cell-by-value* coloring is used. The estimated cellular proportions are shown as decimal numbers next to each cell type (Figure 5D.1). The result indicates that pancreatic islets consist of two rare (circled) and four abundant cell types, and that gene *AP006222.2* is selectively expressed in the six cell types.

#### Visualization of spatially resolved single-cell data

Spatially resolved single-cell assay data (e.g. RNA-seq; Figure 5E) preserve the location of cells within the corresponding source features, such as tissues (5,59). To plot this data, *spatialHeatmap* projects (overlays) the spatially resolved single-cell or population data onto the corresponding features in anatomical images. The resulting plots are referred to as single-cell SHMs (scSHM, Figure 5E.2). Figure 5E gives the co-visualization of spatial single-cell (10X Visium) and bulk (52) RNA-seq data of the sagittal mouse brain. The gene *Efhd2* is spatially enriched in cerebral cortex, which is detected by the *Spatial Enrichment* function. Figure 5E.3 gives the corresponding SHM. Before co-visualization, the two types of data are jointly normalized (e.g. with *Seurat*), then the spatial single-cell data are clustered (18–21). Four of the obtained clusters correspond anatomically to cerebral cortex. Figure 5E.2 is the scSHM of the four clusters colored by the *cell-by-value* scheme, where each cell is positioned according to its spatial coordinates. These cells are also shown in the embedding plot (Figure 5E.1) with the same coloring scheme. The grey dots in Figure 5E.1-2 are cells belonging to other clusters. Figure 5E.2 reveals that *Efhd2* has high and low expression levels in sub-features of cerebral cortex, respectively. These sub-features roughly form two clusters (circled) in Figure 5E.1. This result may indicate that there are potentially novel cell types present in the cerebral cortex.

### Shiny App

**Figure 6.**
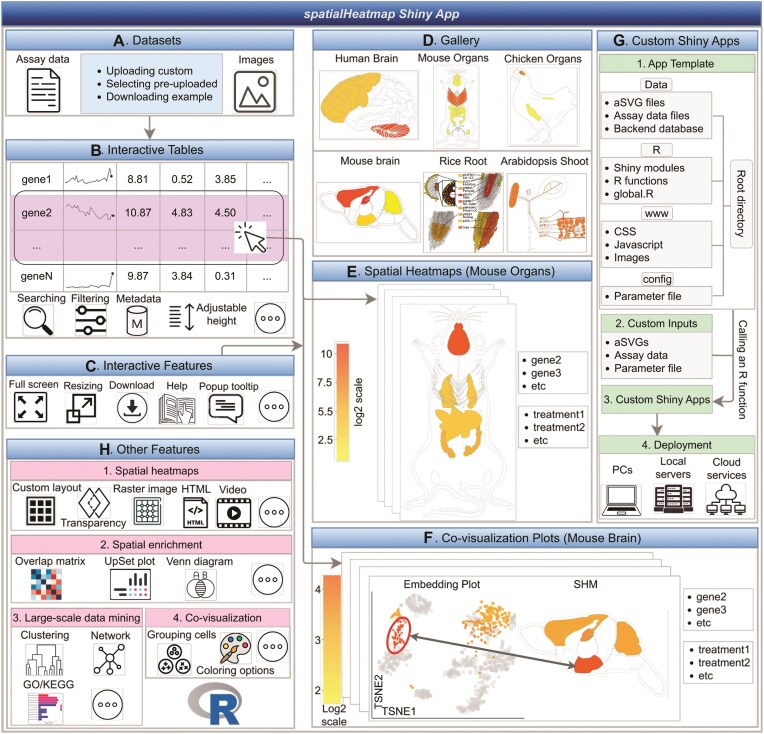
Schematic summary of the *spatialHeatmap* Shiny App. (**A**) The input datasets can be selected from pre-configured examples or uploaded by the user. (**B**) The assay data are presented in interactive tables, that can be searched and filtered. (**C**) Visual representation of interactive features. (**D**) The landing page showcases a gallery of SHMs that can be created within a deployed web instance. (**E, F**) Examples of SHM and single-cell co-visualization plots that can be created by simple click actions in the interactive table. (**G, H**) Overview of additional features.

The Shiny App of *spatialHeatmap* (Figure 6) provides an easy to use GUI that supports most of the functionalities of the R package. A pre-configured live web instance of the Shiny App has been deployed for testing (https://spatialheatmap.org/). The input files and data structures used by the Shiny App are the same as for the R package. This includes bulk and single-cell assay data, and aSVG images. These are described above and illustrated in Figure 2. Important features of the Shiny App are highlighted in Figure 6. For easy testing, sample data are provided in the testing instances (Figure 6A). In addition, users can upload their custom datasets. The Shiny App is divided into two main components or tracks, one for plotting SHMs (Figure 6E) and one for co-visualizing bulk and single-cell data (Figure 6F). A very powerful feature of the Shiny App is that the assay data are available in an interactive table (Figure 6B) where users can create SHMs for biomolecules (e.g. transcripts) simply by selecting the corresponding rows in their assay table. Biomolecules of interest can be identified in the table with search and filter functionalities applied to annotation or numeric fields, respectively. After selecting biomolecules, the corresponding SHMs can be generated and arranged in the plotting space based on default or custom parameters (Figure 6H.1). The other plotting functionalities outlined in Figure 3 are also supported, including transparency and photorealistic effects. In addition to the utilities provided by *spatialHeatmap’s* R package (Figure 6H), the Shiny App provides various unique features (Figure 6B, C). First, reactive tables simplify the generation of complex SHMs and data mining plots for biomolecules of interest, that can be generated by simple mouse over and click actions (Figure 6B). Second, interactive plots provide convenient options for modifying plots or retrieving additional information about data points. Third, several download functions are available to take snapshots of tabular and image analysis results. Fourth, toggling between normal, full screen and scaling modes add clarity in visual analysis routines. Fifth, a dashboard is available for organizing diverse SHMs by assay or anatomical categories (Figure 6D). Sixth, popup tooltips are provided throughout the analysis process along with status reports. Seventh, public Shiny App instances can be created and hosted by research groups to efficiently share their own spatial assay data with the community (Figure 6G.1-3) (60). Eighth, creation and deployment of customized Shiny App instances is simplified by providing a partially automated setup process where complex parameter selections can be specified in a template file (Figure 6G.4). Ninth, Shiny modules are used to maximize reusability and extendability of the system (Figure 6H).

### Use case

**Figure 7.**
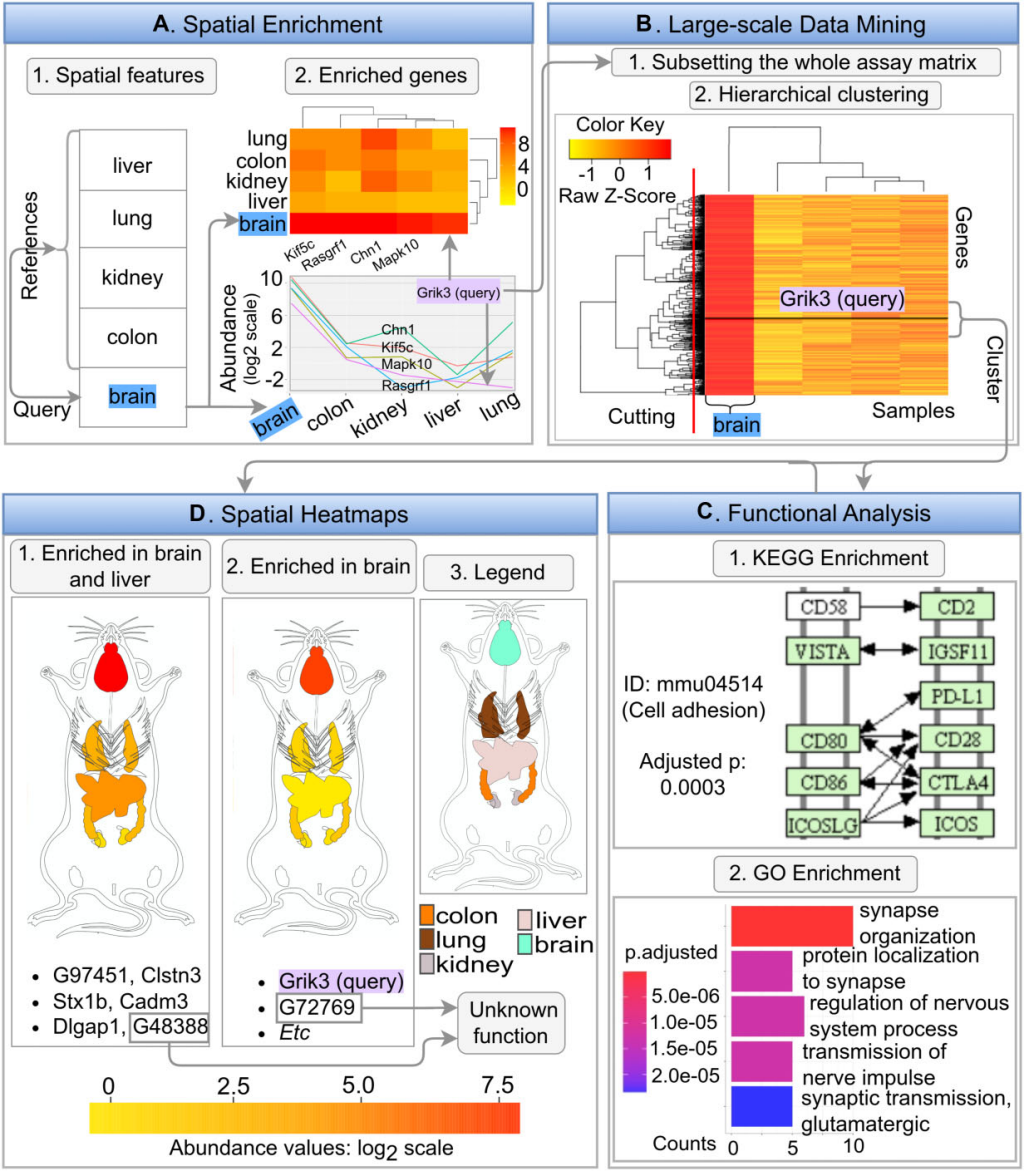
Using *spatialHeatmap* for biological discovery. (**A**) The *Spatial Enrichment* function identifies genes that are selectively expression in brain. Among them is *Grik3*, a well characterized gene involved in neurodegenerative diseases and cancers. (**B**) Genes with similar expression profiles as *Grik3* are identified by querying the expression matrix with a correlation search. After subsetting the expression matrix to the most similar members, hierarchical clustering is performed and the results are visualized in a matrix heatmap. By cutting the row dendrograms (red line) the *Grik3* cluster is identified. (**C**) KEGG and GO term enrichment analysis is performed on the genes of the *Grik3* cluster. (**D**) The spatial expression patterns of the genes in the *Grik3* cluster are analyzed with SHM plots.

*spatialHeatmap* offers functionalities for analyzing spatial assay data with SHMs, and optionally extending them with large-scale data mining routines and integrating single-cell data. The following use case example demonstrates the advantages of these utilities within a discovery workflow (Figure 7). A typical analysis can start with a biomolecule or feature of interest. In the given use case a gene and a tissue are used as specific query examples, and the bioassay data are from an RNA-seq experiment from mouse (47). First, after selecting or importing a spatial bioassay dataset along with a matching anatomical aSVG image of interest (here from EBI’s anatomogram), the user can identify transcripts that are enriched in a chosen query tissue using the *Spatial Enrichment* functionality from *spatialHeatmap*. Second, the expression profiles of the enriched transcripts can be visualized by heat coloring the spatial features in the chosen anatomical image in the form of an SHM plot. An analysis workflow with a known query gene would often start at this SHM plotting step. Third, transcripts with similar expression profiles across a custom selection of samples can be identified with a profile search (e.g. correlation-based search; Figure 7B.1). Third, clustering (Figure 7B.2) and network analysis can be applied to further sub-categorize transcripts by more stringent co-expression requirements. Fourth, the corresponding genes in clusters or network modules obtained can be functionally characterized with pathway and GO enrichment analysis (Figure 7C). Since genes with similar expression patterns are often members of the same molecular or biological processes, it is reasonable to hypothesize that the functionally uncharacterized members within a cluster share similar functions as the characterized members. These insights can be used for assigning hypothetical functions to unknown genes and designing downstream experiments for characterizing gene functions. In the first step that uses *Spatial Enrichment* (Figure 7A), brain is chosen as the spatial feature of interest, and liver, kidney, lung, and colon as the reference features (Figure 7A.1). A variety of plotting options can be used for visualizing spatially-enriched genes. In Figure 7A.2 the genes enriched in brain tissue are plotted in a heatmap as well as a line graph. Among these genes, the expression profile of *Grik3* is chosen as the query. This gene is interesting since it codes for a well-known excitatory neurotransmitter receptor that is involved in the formation of various neurodegenerative diseases and cancers (61,62). To identify genes with highly similar expression profiles, a correlation search (here Pearson’s correlation coefficient) is performed using *Grik3* as the query. After subsetting the assay matrix to the 15% most similar members from this correlation search (including the query gene *Grik3*), hierarchical clustering is performed. The clustering results are presented in the form of a matrix heatmap where the rows and columns are sorted by the dendrograms obtained from the hierarchical clustering step (Figure 7B.2). The gene set of the cluster containing the query gene (named *Grik3* cluster, Supplementary Table S3) is identified by applying a tree cutting algorithm to the gene dendrogram. To functionally annotate the obtained gene cluster, functional enrichment analysis of KEGG and GO terms is performed. The result shows that the *Grik3* cluster is enriched in genes involved in cell adhesion and synapse-related activities, respectively (Figure 7C). This agrees well with the fact that *Grik3* is a well-characterized neurotransmitter receptor in the plasma membrane (62). Next, SHM plotting is used to inspect the spatial expression patterns of the 36 genes in the *Grik3* cluster (Figure 7D). The results in Figure 7D indicate that the genes of the *Grik3* cluster are expressed at higher levels in brain than in the other organs considered in the SHMs. In addition, six of these genes are also relatively highly expressed in liver (Figure 7D.1). In contrast to this, all other genes of the same cluster are exclusively enriched in brain (Figure 7D.2). Based on the above functional enrichment results of this cluster and the known involvement of several members in neurological disease and cancer development, including *Cdk5r1, Dlgap1, Stx1b, Scn8a and Nrxn1* (63–68), it is reasonable to hypothesize that the genes of this cluster are part of a more complex disease pathway. Their investigation could provide novel insights into the common mechanisms of these complex diseases. Furthermore, the biological functions of *G48388* and *G72769* are still unknown. Their co-clustering with the known genes of the *Grik3* cluster suggests they may have a similar biological function, and are potentially involved in neurological diseases and different types of cancer (69). This use case example showcases the usefulness of the visualization and data mining utilities implemented in *spatialHeatmap*.

## Discussion

### Significance

Living organisms are characterized by a high degree of structural organization at the cell, tissue and organ level. Spatial biology aims to uncover the mechanisms that govern the development and evolution of these structures using both experimental and quantitative methods. Characterizing these complex processes with high spatiotemporal resolution is critical for understanding how cells, tissues and organisms develop, interact and respond to their environment.

Recently introduced large-scale spatial profiling technologies, such as conventional and spatially resolved single-cell transcriptomics and proteomics (5,59,70–74), have substantially increased the resolution of spatial assays down to the cellular and subcellular levels. These high resolution spatial profiling technologies have enormous potential of transforming our understanding of biological processes. The resulting insights have many applications in basic and translational research in agriculture and biomedical research. In addition, evolutionary biology can study what role spatially selective processes play in the adaptation of organisms.

### Enhancements to spatial biology

Efficient processing of spatial data requires new software for analyzing, modeling and visualizing the complex data generated by spatial biologists. The *spatialHeatmap* software contributes to the spatial visualization field by providing a series of useful functionalities for plotting and intuitively interpreting spatial assay data. A high degree of customizability allows users to work with custom image and assay data obtained from their own experiments, or from public repositories.

A semi-automated layout feature facilitates systematic comparisons among large numbers of SHMs, that are often required for analyzing spatial abundance trends of biomolecules from assay data with complex experimental designs. This feature is particularly useful for deriving insights into spatial abundance dynamics across multiple biomolecules (e.g. chromatin, mRNA, proteins or metabolites) or a series of treatments, such as treatments with different stresses, pathogens, genetic or small molecule perturbations, and time-series experiments. It enables researchers to identify novel connections among spatial patterns frequently observed in different developmental stages, responses to stresses or diseases. Another new feature is the option to create photo-realistic SHMs by superimposing SHM line drawings onto raster graphics, while an adjustable transparency option maintains visual clarity in the resulting composite image. As a result, additional structural details can be shown in SHMs, such as locations of organelles or vesicles within the cells of a given tissue. A similar transparency mechanism is used for viewing overlapping spatial features in layered SVG images. With this, features in lower layers can be visually exposed by making the features in upper layers transparent. Support for comparative SHM analysis across species is another distinct feature. Spatial patterns only observed in certain species but absent in others can be associated with traits or phenotypes a researcher is interested in.

Apart from plotting SHMs, several large-scale data mining extensions are integrated into the *spatialHeatmap* that enable users to identify for a query biomolecule other biomolecules with similar abundance profiles. This is achieved with pattern searching, clustering and network construction functionalities built into the environment. The obtained clusters and network modules can be functionally characterized with GO term and pathway enrichment tools.

Comparative analysis of spatial bulk and single-cell data is provided by a new co-visualization feature that integrates the two data types. This opens new ways for analyzing spatial omics data and planning the corresponding experiments. Often it is only possible to generate omics data for large numbers of experimental variables with bulk approaches as the corresponding single-cell experiments would be too costly and complex. With the availability of a co-visualization tool, one can generate single-cell data for smaller numbers of treatments, and then project or predict the remaining ones from the bulk space to the single-cell data space using various strategies including deconvolution algorithms (27,53–56). Currently, the co-visualization module of *spatialHeatmap* co-visualizes bulk data with standard or spatially resolved single-cell data (Figure 5E), and to tentatively associate single cells with source tissues via co-clustering. The combined plots facilitate the visual analysis of single-cell embedding plots in the context of the corresponding source tissues. This is useful for a series of applications. Some of the most important ones include: (1) associating unlabeled single cells with tissues and organs based on co-expressed marker genes; (2) detecting potentially mislabelled cells when the co-expression results disagree with each other; (3) predicting spatial sub-regions or functional specializations in tissues based on characteristic cell sub-clusters visible in the embedding plots; and (4) identifying common cell types present in distinct tissues (e.g. vascular cells). *spatialHeatmap’s* co-visualization module is designed to accommodate the rapidly expanding data modalities of cellular spatial biology, including more economic deconvolution designs (26–28,53–56) or spatially resolved single cell data.

### Reusable and extendable software design

The data structures in *spatialHeatmap* are designed with sufficient abstraction to maximize generalization, reusability and efficiency. This includes as much as possible adaptation of widely used community data structures, such as *SE* and *SCE* (30), two of the core classes for handling bulk and single-cell assay data provided by the Bioconductor project, where *spatialHeatmap* is hosted. Only when needed, new data containers are introduced, such as a new S4 *SVG* class for lossless import/export and efficient handling of SVG image files. SVG was chosen as the core vector-based image format, because of its wide adaptation as a community standard as well as its computational accessibility (details discussed in Materials and methods). Since the SVG format is ideal for creating custom illustrations and its wide usage, users have the flexibility to work with their own custom SVG images or download them from collections from several spatial data providers, such as the Expression Atlas, BAR or EBI anatomogram (6,7,12,75).

*spatialHeatmap* is an R package with a GUI extension implemented as a Shiny App. The R package is the backend of the software environment that can be used in command-driven mode from within R, while the Shiny App provides web browser-based access to the same backend. This design has many advantages. First, it serves both computational and experimental scientists by supporting analysis needs requiring programmable or interactive access, respectively. Second, development of all core functionalities within R makes deployment of the corresponding features in the Shiny App relatively straightforward and uniform. Third, initialization of Shiny App instances is flexible and straightforward, even for unexperienced users, as they can run on personal computers, web servers or cloud-based systems. Fourth, a wider range of functionalities can be provided when combining a command-driven with a graphical interface as certain functionalities are only available in either one of them. For instance, the R interface supports a higher level of reproducibility via programmable solutions. On the other hand, modern web technologies offer powerful visual data mining features. Access to these is provided via the Shiny App, such as highly interactive tables and graphics. Fifth, the option to easily deploy custom web instances of *spatialHeatmap* allows research groups to openly share their spatial omics data. This addresses the urgent need of sharing spatial research data with the community in ways that elevate the transparency and accessibility of large scale omics data. Combined, the functionalities integrated in *spatialHeatmaps* provide a novel toolbox for spatial data analysis and visualization.

### Future work

In the future we will continue to enhance *spatialHeatmap* by adding several new features. Currently, the environment is optimized for viewing single- or multi-dimensional data of a single data modality, such as plotting in a single window either transcriptomics, proteomics or metabolomics data, but not multiple data types at the same time. Support for simultaneous viewing of multi-omics experiments will facilitate comparative analysis needs across multi-modal data. This will be achieved by organizing multi-omics data in Bioconductor’s *MultiAssayExperiment* (76) data container that is optimized for efficient handling and harmonizing multi-omics assay data. For comparative visual analysis of multi-omics data, SHMs obtained from different data modalities will be displayed in separate panels within a single window view. This new functionality will facilitate the identification of spatial abundance patterns that are preserved across different data types or specific to individual ones.

Additional supervised and non-supervised large-scale machine learning methods will be added to the large-scale data mining module. This will include several clustering, network construction and classification methods. More recently developed spatial deconvolution algorithms will also be added, such as *Bulk2Space* (77). The inventory of ready-to-use SVG images will be expanded by adding additional images to the gallery from this project, providing broad support for the SVG variants from other spatial image repositories, as well as working on the standardization of SVG annotations for spatial data sciences.

## Conclusion

*spatialHeatmap* is a new software for visualizing spatial assay data in anatomical images with extensions for large-scale data mining and co-visualization of single-cell data. It integrates many novel functionalities to a generic spatial visualization toolbox that is flexible, extensible, and object-oriented by design. Graphical and command-line interfaces are provided to support the needs of both non-expert and computational users.

## Supplementary Material

lqae006_Supplemental_File

## Data Availability

*spatialHeatmap* is an open source package that has been reviewed, tested and accepted by the Bioconductor project. It is freely available for all common operating systems from Bioconductor and GitHub here: https://doi.org/doi:10.18129/B9.bioc.spatialHeatmap. The DOI is: https://doi.org/doi:10.18129/B9.bioc.spatialHeatmap.
