# Supplementary material for: spatialHeatmap: visualizing spatial bulk and single-cell assays in anatomical images
Source: NAR Genom Bioinform. 2024 Feb 2;6(1):lqae006. doi: 10.1093/nargab/lqae006 (PMC10836942; doi:10.1093/nargab/lqae006)
Supplement: lqae006_Supplemental_File [file lqae006_supplemental_file.pdf]

# SUPPLEMENTARY MATERIAL

## SUPPLEMENTARY SECTIONS

### S1

### Network analysis

At the time of writing, *spatialHeatmap* uses WGCNA for network analysis (1). This approach includes five major steps. First, a correlation matrix is computed from the numeric values of items considered in an analysis (*e.g.* abundance or fold changes of genes or other biomolecules). Second, the obtained matrix is transformed into an adjacency matrix defining the connections among items. Third, the adjacency matrix is used to calculate a topological overlap matrix (*TOM*) where shared neighborhood information among items is used to preserve robust connections while removing spurious connections. Fourth, the distance-transformed TOM is used for hierarchical clustering. To maximize time performance, the latter is performed with the *flashClust* package (2). Fifth, network modules are identified with the *dynamicTreeCut* package (3). The stringency for identifying modules can be controlled with the *ds* (*deepSplit*) argument. The result is a *list* containing the adjacency matrix and the final module assignments stored in a *data.frame*. Because the interactive visualization of the network graph performs best on smaller modules, the Shiny App of *spatialHeatmap* returns by default only modules that were obtained with relatively stringent settings.

### S2

### Co-clustering of bulk and single-cell data

The co-clustering method of bulk and single-cell data is illustrated in Figure S1. This method aims to computationally assign tissue labels to individual cells in cases where the single-cell data are entirely or partially unlabeled. Subsequently, the obtained tissue-to-cell labels can be utilized in co-visualization plots for coloring single cells by the predicted source tissues. To obtain meaningful results with this method, the single-cell and bulk expression data should be from matching or at least comparable tissues. Since single-cell data usually have lower sensitivity and higher sparsity than bulk data, the below adjustment steps were applied to make the single-cell data more comparable to the bulk data. To maximize the accuracy of the predictions, the method has been optimized on real data with known tissue-to-cell assignments. The details of this optimization are available in *spatialHeatmap's* co-visualization vignette. The following outline of this method uses RNA-Seq data as an example. Data from other profiling technologies can be used as well. First, the bulk and single-cell data are joined into a single matrix $m$ where rows and columns are biomolecules (*e.g.* genes) and samples (here cells and bulk tissues), respectively (Figure S1A). Next, $m$ is pre-processed by applying normalization and filtering routines (4). The filtering subsets the rows and columns in $m$ to user-selectable proportions of biomolecules and cells, respectively, without removing the columns of the bulk tissue samples. This filtering reduces the sparsity of the single-cell data, while making them more comparable with the bulk tissue data. Second, the column data are embedded with dimension reduction algorithms (4), such as PCA or UMAP (Figure S1B). Third, co-clustering is performed on the embedding data. Specifically, a graph is built on the top joint dimensions using methods (*buildKNNGraph* or *buildSNNGraph*) from *scran* where nodes are cells (or tissues) and edges are connections between nearest neighbors (5), and subsequently this graph is partitioned with methods (*cluster_walktrap*, *cluster_fast_greedy*, or *cluster_leading_eigen*) from *igraph* to obtain clusters (6), which correspond to groups of cells and tissues that exhibit highly similar expression patterns. The choice of the methods and their parameters were optimized on test data with known cell-to-tissue assignments. In the example illustrated in Figure S1C, three types of clusters are shown: (i) multiple cells are co-clustered and assigned to one bulk tissue sample; (ii) multiple cells are co-clustered with several bulk tissues, and then assigned to a single bulk tissue with a nearest-neighbor approach; and (iii) cells that do not co-cluster with any bulk tissue remain unassigned. Fourth, after co-clustering, cells are labeled by bulk tissues or remain un-labeled (Figure S1D). Fifth, the obtained labels are subsequently used to match cells with tissues in embedding and SHM plots, respectively (Figure S1E).

### S3

This supplement section contains additional supporting material for examples in the *Results* section.

**Table S1.** Cluster containing the query gene *Ugp2* in the hierarchical clustering example.

| | Ensembl id | Uniprot symbol | Entrez id |
|---|---|---|---|
| 1 | ENSMUSG00000001891 | Ugp2 (query) | 216558 |
| 2 | ENSMUSG00000010025 | Aldh3a2 | 11671 |
| 3 | ENSMUSG00000015714 | Cers2 | 76893 |
| 4 | ENSMUSG00000015846 | Rxra | 20181 |
| 5 | ENSMUSG00000017009 | Sdc4 | 20971 |
| 6 | ENSMUSG00000018677 | Slc25a39 | 68066 |
| 7 | ENSMUSG00000020091 | Eif4ebp2 | 13688 |
| 8 | ENSMUSG00000020741 | Cluh | 74148 |
| 9 | ENSMUSG00000021000 | Mia2 | 338320 |
| 10 | ENSMUSG00000021236 | Entpd5 | 12499 |
| 11 | ENSMUSG00000022214 | Dcaf11 | 28199 |
| 12 | ENSMUSG00000022982 | Sod1 | 20655 |
| 13 | ENSMUSG00000024507 | Hsd17b4 | 15488 |
| 14 | ENSMUSG00000024953 | Prdx5 | 54683 |
| 15 | ENSMUSG00000025950 | Idh1 | 15926 |
| 16 | ENSMUSG00000026385 | Dbi | 13167 |
| 17 | ENSMUSG00000028127 | Abcd3 | 19299 |
| 18 | ENSMUSG00000028405 | Aco1 | 11428 |
| 19 | ENSMUSG00000031770 | Herpud1 | 64209 |
| 20 | ENSMUSG00000032047 | Acat1 | 110446 |
| 21 | ENSMUSG00000047866 | Lonp2 | 66887 |
| 22 | ENSMUSG00000049422 | Chchd10 | 103172 |
| 23 | ENSMUSG00000058135 | Gstm1 | 14862 |

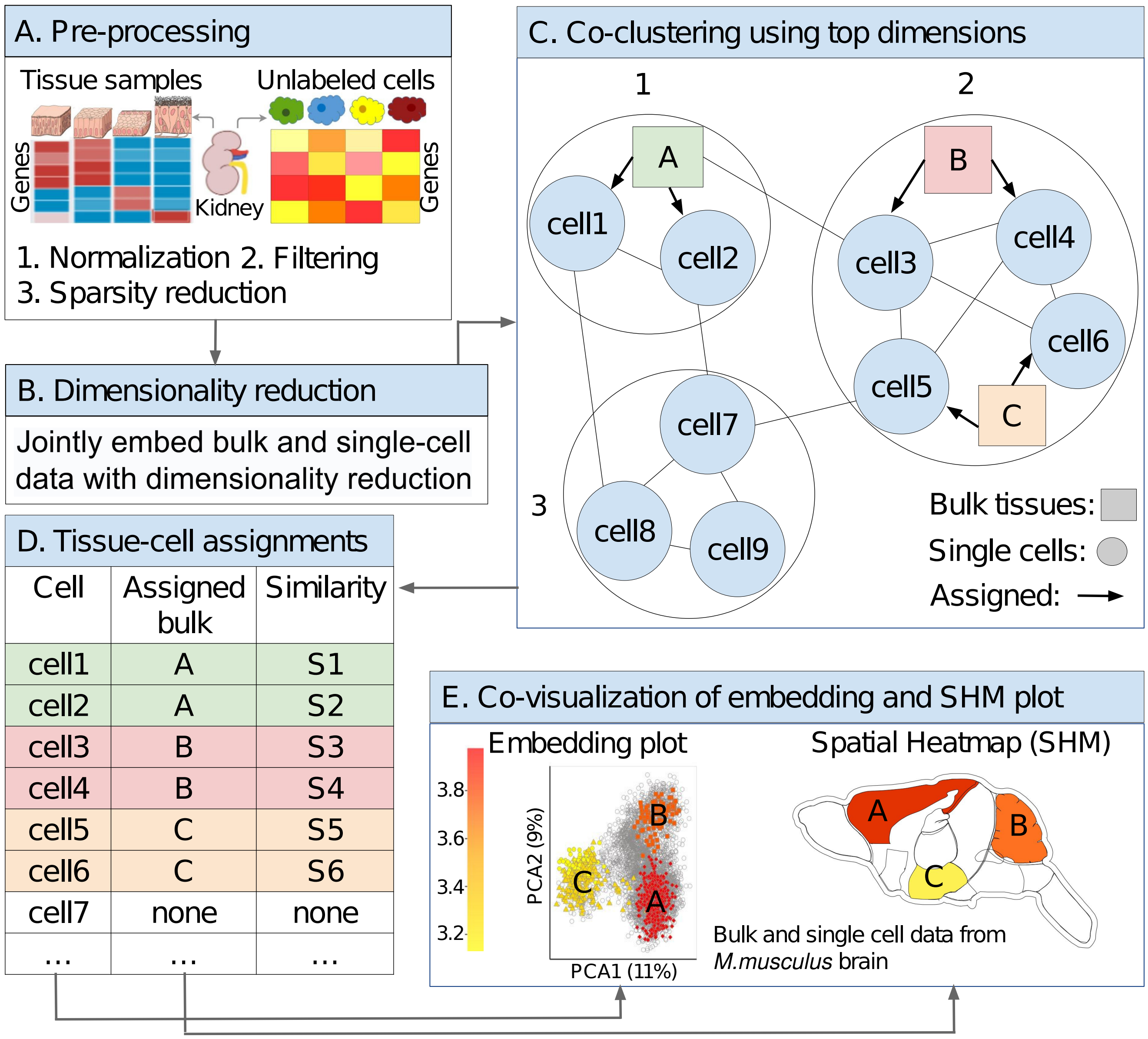


**Figure S1.** Co-clustering illustration. **(A)** The single-cell and bulk tissue data are jointly pre-processed. **(B)** Single-cell and bulk data are embedded with dimension reduction methods. **(C)** The embedding results are used for co-clustering single-cells and bulk tissue data. Cells are assigned to tissues based on the clustering results as follows: (1) If a cluster contains a single tissue, then the cells of this cluster are assigned to the corresponding tissue. (2) If a cluster contains multiple tissues and cells, a nearest-neighbor approach resolves this ambiguous situation by assigning cells to the closest tissue sample. (3) Cells in clusters without tissue samples remain unassigned. **(D)** The cell-tissue assignments and the similarity scores of the predictions are stored in a table. **(E)** The predictions can be used to color the cells by predicted source tissues in co-visualization plots.

**Table S2.** Network module containing the query gene *Serpina1b* in the network analysis example.

| | Ensembl id | Uniprot symbol | Entrez id |
|---|---|---|---|
| 1 | ENSMUSG00000017344 | Vtn | 22370 |
| 2 | ENSMUSG00000025271 | Pfkfb1 | 18639 |
| 3 | ENSMUSG00000038539 | Atf5 | 107503 |
| 4 | ENSMUSG00000041798 | Gck | 103988 |
| 5 | ENSMUSG00000045094 | Arhgef37 | 328967 |
| 6 | ENSMUSG00000048856 | Slc25a47 | 104910 |
| 7 | ENSMUSG00000071178 | Serpina1b | 20701 |
| 8 | ENSMUSG00000092021 | | 634650 |
| 9 | ENSMUSG00000109764 | Klkb1 | 16621 |

**Table S3.** Cluster containing the query gene *Grik3* in the use case example.

| | Ensembl id | Uniprot symbol | Entrez id |
|---|---|---|---|
| 1 | ENSMUSG00000001985 | Grik3 (query) | 14807 |
| 2 | ENSMUSG00000003273 | Ca11 | 12348 |
| 3 | ENSMUSG00000003279 | Dlgap1 | 224997 |
| 4 | ENSMUSG00000005338 | Cadm3 | 94332 |
| 5 | ENSMUSG00000008153 | Clstn3 | 232370 |
| 6 | ENSMUSG00000019831 | Wasf1 | 83767 |
| 7 | ENSMUSG00000020333 | Acsl6 | 216739 |
| 8 | ENSMUSG00000020684 | Rasl10b | 276952 |
| 9 | ENSMUSG00000022523 | Fgf12 | 14167 |
| 10 | ENSMUSG00000023033 | Scn8a | 20273 |
| 11 | ENSMUSG00000024109 | Nrxn1 | 18189 |
| 12 | ENSMUSG00000024524 | Gnal | 14680 |
| 13 | ENSMUSG00000024873 | Cnih2 | 12794 |
| 14 | ENSMUSG00000025272 | Tro | 56191 |
| 15 | ENSMUSG00000025427 | Rnf165 | 225743 |
| 16 | ENSMUSG00000025576 | Rbfox3 | 52897 |
| 17 | ENSMUSG00000025876 | Unc5a | 107448 |
| 18 | ENSMUSG00000026442 | Nfasc | 269116 |
| 19 | ENSMUSG00000030683 | Sez6l2 | 233878 |
| 20 | ENSMUSG00000030806 | Stx1b | 56216 |
| 21 | ENSMUSG00000032773 | Chrm1 | 12669 |
| 22 | ENSMUSG00000032890 | Rims3 | 242662 |
| 23 | ENSMUSG00000033597 | Caskin1 | 268932 |
| 24 | ENSMUSG00000036634 | Mag | 17136 |
| 25 | ENSMUSG00000038555 | Reep2 | 225362 |
| 26 | ENSMUSG00000043419 | Rnf227 | |
| 27 | ENSMUSG00000048388 | | 241520 |
| 28 | ENSMUSG00000048895 | Cdk5r1 | 12569 |
| 29 | ENSMUSG00000050587 | Lrrc4c | 241568 |
| 30 | ENSMUSG00000053693 | Mast1 | 56527 |
| 31 | ENSMUSG00000053825 | Ppfia2 | 327814 |
| 32 | ENSMUSG00000059974 | Ntm | 235106 |
| 33 | ENSMUSG00000069806 | Cacng7 | 81904 |
| 34 | ENSMUSG00000071862 | Lrrtm2 | 107065 |
| 35 | ENSMUSG00000072769 | | |
| 36 | ENSMUSG00000097451 | | |

## REFERENCES


1. Langfelder, P. and Horvath, S. (December, 2008) WGCNA: an R package for weighted correlation network analysis. *BMC Bioinformatics,* **9**, 559.
2. Langfelder, P. and Horvath, S. (2012) Fast R Functions for Robust Correlations and Hierarchical Clustering. *Journal of Statistical Software,* **46**(11), 1–17.
3. Langfelder, P., Zhang, B., and Horvath, S. (March, 2008) Defining clusters from a hierarchical cluster tree: the Dynamic Tree Cut package for R. *Bioinformatics,* **24**(5), 719–720.
4. McCarthy, D. J., Campbell, K. R., Lun, A. T. L., and Willis, Q. F. (2017) Scater: pre-processing, quality control, normalisation and visualisation of single-cell RNA-seq data in R. *Bioinformatics,* **33**, 1179–1186.
5. Lun, A. T. L., McCarthy, D. J., and Marioni, J. C. (2016) A step-by-step workflow for low-level analysis of single-cell RNA-seq data with Bioconductor. *F1000Res.,* **5**, 2122.
6. Csardi, G. and Nepusz, T. (2006) The igraph software package for complex network research. *InterJournal,* **Complex Systems**, 1695.
